# Skin Lesions of Disseminated Histoplasmosis Resembling Kaposi’s Sarcoma

**DOI:** 10.4269/ajtmh.22-0459

**Published:** 2023-01-23

**Authors:** Akira Kawashima, Haruka Uemura, Shinichi Oka

**Affiliations:** AIDS Clinical Center, National Center for Global Health and Medicine, Tokyo, Japan

**Figure 1.**
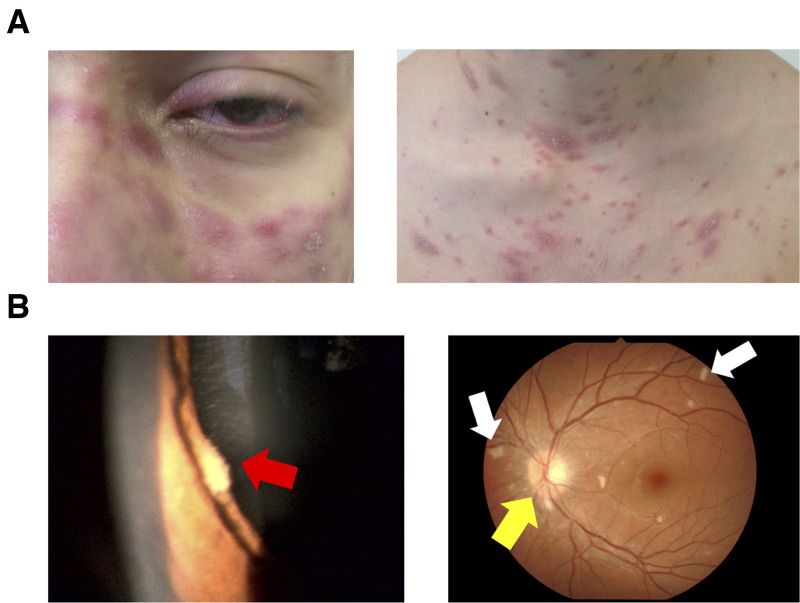
(**A**) Generalized erythema with partial crusting was noted on the patient’s face, trunk, and limbs. The lesions resembled Kaposi’s sarcoma. (**B**) An ophthalmic examination performed 2 weeks after admission revealed iris nodules (red arrow), papillary edema (yellow arrow), and hard white exudates (white arrows).

**Figure 2.**
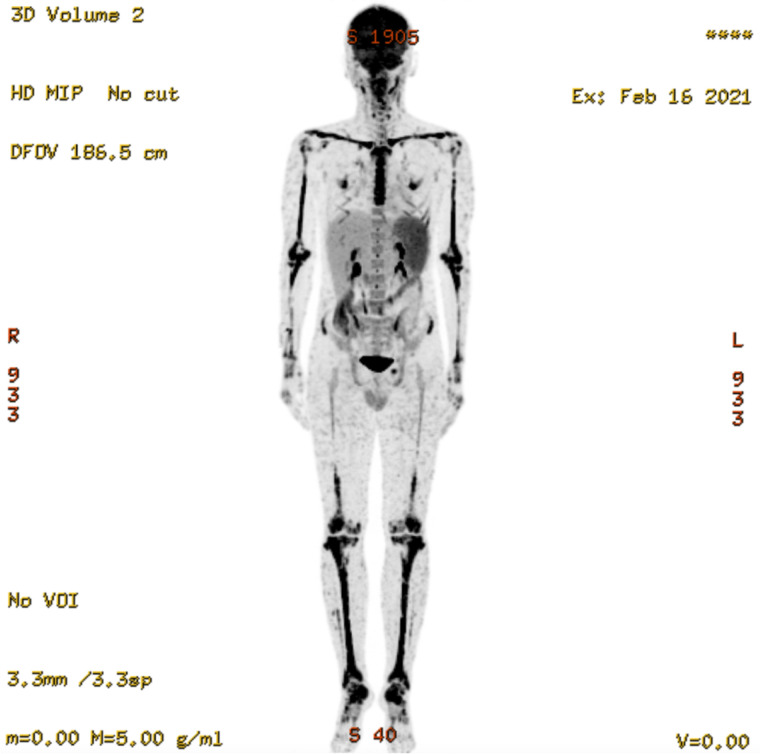
Positron emission tomography scan showing fluorine-18 deoxyglucose accumulation in multiple subcutaneous nodules, the spleen, bone marrow, and bones throughout the body.

A 29-year-old man with newly diagnosed HIV infection and a 6-week history of recurrent fever, weight loss, and rashes was referred to our hospital. He had immigrated to Japan from Brazil 15 years previously. Physical examination revealed a body temperature of 40.2°C and generalized erythema with partial crusting on his face, trunk, and limbs (Figure 1A). Laboratory tests showed a white blood cell count of 3.54 × 10^9^ cells/L, CD4 count of 21 cells/μL, hemoglobin of 9.5 g/dL, aspartate aminotransferase of 133 U/L, alanine aminotransferase of 108 U/L, lactate dehydrogenase of 948 U/L, serum creatinine of 0.69 mg/dL, and β-d-glucan level of 164.8 pg/mL. A polymerase chain reaction (PCR) test for human herpes virus 8 (HHV-8) was negative. Ophthalmic examination revealed iris nodules, papillary edema, and hard white exudates (Figure 1B). Positron emission tomography scan showed fluorine-18 deoxyglucose accumulation in multiple subcutaneous nodules and the spleen, bone marrow, and bones (Figure 2).

**Figure 3.**
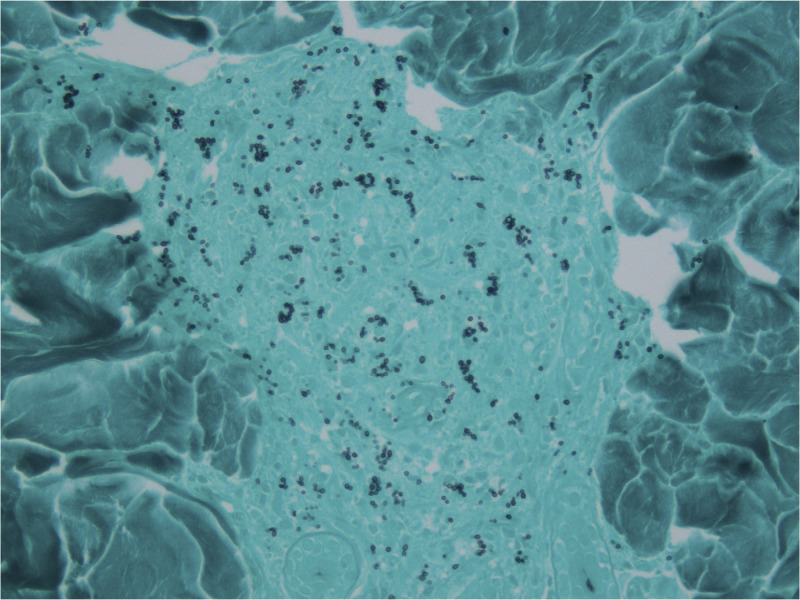
A skin biopsy was performed, and the specimens were stained with Grocott methenamine silver stain (magnification ×400).

Although the skin lesions resembled those of Kaposi’s sarcoma, which are not usually biopsied per our protocol, the fever, positive β-d-glucan result, and negative HHV-8 result led us to suspect systemic mycosis. Skin biopsy and bone marrow aspiration were performed, and the specimens were stained with Grocott methenamine silver (Figure 3). The patient was diagnosed with disseminated histoplasmosis (DH) on the basis of PCR and cultures obtained from the skin and bone biopsy specimens.

Histoplasmosis is caused by *Histoplasma capsulatum*, which is endemic in North, Central, and South America, but not in Japan. As seen in this case, *H. capsulatum* can remain dormant in the body for over 10 years.^1^ The patient’s country of origin increased our suspicion for this disease. Disseminated histoplasmosis is an opportunistic infection affecting immunocompromised persons, such as those with HIV.^2^ It is a progressive extrapulmonary infection that undergoes hematogenous dissemination to the bone marrow, liver, spleen, and lymph nodes,^2^ and common cutaneous manifestations include papules and oral erosions.^3^

The treatment of HIV-positive DH involves liposomal amphotericin B for at least 2 weeks or until clinical improvement.^2^ Antifungal drugs are immediately followed by anti-retroviral therapy (ART).^2^ Iritis and optic neuritis have been reported in autopsies,^4^ and blindness due to immune reconstitution inflammatory syndrome (IRIS) was reported.^5^ Therefore, our patient was treated with liposomal amphotericin B (3 mg/kg) for 4 weeks without ART until the cutaneous and ocular symptoms improved. Two weeks after ART initiation with coformulated bictegravir, emtricitabine, and tenofovir alafenamide, the patient showed no signs of IRIS; therefore, his antifungal treatment was switched to itraconazole (200 mg twice daily). One year after discharge, he had not experienced a relapse.
